# 

**Published:** 1888-07

**Authors:** 


ORIGINAL COMMUNICATIONS:

Remarks on Pelvic Inflammations, and the Management of their Residues. By Wm. Warren Potter, M. D., . . . . . . . . 530
The Electro-Magnet in Removal of Steel from the Interior of the Eye. By Alvin A. Hubbell, M. D., . . . . . . . . . 545
Art of Infant Feeding. By W. J. Thayer, D. D. S., M. D., . . . . . . . . . . 554

MISCELLANY:

Medical Aphorisms, . . . . . . . . . 558
Pharmacy in the Sixteenth Century, . . 559
Is Consumption Contagious, . . . . . 559
Honors to von Brucke, . . . . . . . 560
The Suppression of Quack Medicine in Germany, . . . . . . . . . . . . 560

SELECTIONS:

On Leucocythæmia, considered as Specific Cancer of the Blood. By L. Bard, . . 561

Naupathia, . . . . . . . . . . . . . 563

Cascara Sagrada in Rheumatism. By H. T. Goodwin, M. D., . . . . . . . . . 566

EDITORIAL:

In Memoriam, . . . . . . . . . . . . 568
Medical Department of Niagara University, 569
Dr. O'Brien's Nomination for the Position of Health Officer of the Port of New York, . . . . . . . . . . . . . 571
Quack Advertisements in the Religious Press, . . . . . . . . . . . . . 572

BOOK REVIEWS:

The Pathology, Diagnosis and Treatment of the Diseases of Women. By Grailey Hewitt, M. D., . . . . . . . . . . . 575
The Physician's Leisure Library, . . . . 575
Transactions of the New York State Medical Association for the Year 1887. Edited by Alfred L. Carroll, M. D., . . . . . . 576
The Essentials of Medical Chemistry and Urinalysis. By S. E. Woody, A. M., M. D., . . . . . . . . . . . . . 576

